# Persistent Borrelia Infection in Patients with Ongoing Symptoms of Lyme Disease

**DOI:** 10.3390/healthcare6020033

**Published:** 2018-04-14

**Authors:** Marianne J. Middelveen, Eva Sapi, Jennie Burke, Katherine R. Filush, Agustin Franco, Melissa C. Fesler, Raphael B. Stricker

**Affiliations:** 1 Atkins Veterinary Services, Calgary, AB T3B 4C9, Canada; middel@telus.net; 2 Department of Biology and Environmental Science, University of New Haven, West Haven, CT 06516, USA; unh@evasapi.net (E.S.); katherine.r.filush@gmail.com (K.R.F.); 3 Australian Biologics, Sydney, NSW 2000, Australia; Jennie.burke@australianbiologics.com.au; 4 School of Health Sciences, Universidad Catolica Santiago de Guayaquil, Guayaquil 090615, Ecuador; agustin.franco@optusnet.com.au; 5 Union Square Medical Associates, 450 Sutter Street, Suite 1504, San Francisco, CA 94108, USA; melissacfesler@gmail.com

**Keywords:** Lyme disease, *Borrelia burgdorferi*, tickborne disease, chronic infection, spirochete culture

## Abstract

Introduction: Lyme disease is a tickborne illness that generates controversy among medical providers and researchers. One of the key topics of debate is the existence of persistent infection with the Lyme spirochete, *Borrelia* *burgdorferi*, in patients who have been treated with recommended doses of antibiotics yet remain symptomatic. Persistent spirochetal infection despite antibiotic therapy has recently been demonstrated in non-human primates. We present evidence of persistent *Borrelia* infection despite antibiotic therapy in patients with ongoing Lyme disease symptoms. Methods: In this pilot study, culture of body fluids and tissues was performed in a randomly selected group of 12 patients with persistent Lyme disease symptoms who had been treated or who were being treated with antibiotics. Cultures were also performed on a group of ten control subjects without Lyme disease. The cultures were subjected to corroborative microscopic, histopathological and molecular testing for *Borrelia* organisms in four independent laboratories in a blinded manner. Results: Motile spirochetes identified histopathologically as *Borrelia* were detected in culture specimens, and these spirochetes were genetically identified as *Borrelia* *burgdorferi* by three distinct polymerase chain reaction (PCR)-based approaches. Spirochetes identified as *Borrelia burgdorferi* were cultured from the blood of seven subjects, from the genital secretions of ten subjects, and from a skin lesion of one subject. Cultures from control subjects without Lyme disease were negative for *Borrelia* using these methods. Conclusions: Using multiple corroborative detection methods, we showed that patients with persistent Lyme disease symptoms may have ongoing spirochetal infection despite antibiotic treatment, similar to findings in non-human primates. The optimal treatment for persistent *Borrelia* infection remains to be determined.

## 1. Introduction

Lyme disease (LD) and similar Lyme-like *Borrelia* infections are caused by members of the *Borrelia burgdorferi* (Bb) sensu lato complex or by members of the *Borrelia* relapsing fever complex such as *B. miyamotoi*, respectively [1,2,3,4]. Following initial infection, *Borrelia* spirochetes can evade host defenses, sequester in immune privileged sites such as joints or the central nervous system, and persist in pleomorphic forms [5,6,7,8]. Tickborne coinfections including *Babesia*, *Anaplasma*, *Ehrlichia*, *Bartonella* and *Rickettsia* may complicate the clinical picture [6,9,10]. If LD is not treated early in the course of infection, chronic illness may result and a variety of symptoms may develop. These symptoms include fatigue, musculoskeletal pain, arthritis, cardiac disease and neurological involvement with peripheral neuropathy, meningitis, encephalitis, cranial neuritis and cognitive dysfunction [6,8,11,12].

Although LD was first recognized in 1975, it remains a controversial illness and the topic of polemic debate [6,10,13,14,15]. One viewpoint claims that persistent Lyme disease symptoms are related to ongoing spirochetal infection despite antibiotic therapy. This scenario has been demonstrated in animal models including rodents, dogs and horses using various detection methods [16,17,18,19,20,21,22,23,24,25,26,27,28,29,30,31,32,33,34,35,36], and a recent study in non-human primates showing “persistent, intact, metabolically-active *B. burgdorferi* after antibiotic treatment of disseminated infection” offers the strongest support for this pathogenesis [37]. Furthermore, comparable studies have suggested persistent infection after antibiotic therapy as a cause of chronic symptoms in humans [38,39,40,41,42,43,44,45,46,47,48,49,50,51,52,53,54,55,56,57,58,59,60]. The opposing viewpoint claims that persistent Lyme disease symptoms may be due to spirochetal “debris” without active infection. While a number of studies from Europe and the USA have demonstrated persistence of Bb DNA or antigens in human bodily tissues or fluids, very few studies have demonstrated culture of live *Borrelia* spirochetes, the highest form of evidence for persistent infection in chronic Lyme disease patients [4,51,53,59].

In this pilot study, we present detailed evidence of persistent *Borrelia* infection despite antibiotic therapy in 12 randomly-selected North American patients with ongoing LD symptoms. Spirochetal infection was demonstrated by corroborative microscopic, histopathological and molecular detection of live *Borrelia* organisms in cultures of body fluids and tissues from these patients.

## 2. Methods

### 2.1. Subject Selection

Subjects included in the study were chosen at random from our North American patient population. All of the LD patients in the study were either clinically diagnosed with LD or had positive Bb serological testing prior to study participation. Serological testing for LD was performed by a Clinical Laboratory Improvement Amendments (CLIA)-certified laboratory (IGeneX Laboratory in Palo Alto, CA, USA), as described in detail elsewhere [60]. Subjects with Morgellons disease (MD) who were seropositive for LD were included in the study (see below) [61]. All subjects had been treated with antibiotics prior to the study, and symptomatic patients who remained on antibiotic treatment were included in the study.

### 2.2. Control Selection

Ten healthy subjects were recruited as controls after informed consent was obtained. These subjects were then tested serologically for LD and those who were negative were accepted as controls. Vaginal or seminal fluids were collected from negative controls and cultured for *Borrelia*, as described below. Culture pellets underwent PCR testing for *Borrelia* in a blinded manner at the University of New Haven and Australian Biologics, as described below.

### 2.3. Informed Consent

All subjects were adults who gave informed consent to participate in the study. Signed informed consent to collect specimens was obtained in accordance with the ethics approval requirements for sample collection of the Western Institutional Review Board, Puyallup, WA, USA (Study # 1148461). Approval for anonymous sample testing was also obtained from the Institutional Review Board of the University of New Haven, West Haven, CT, USA. Additional signed informed consent to publish the results was obtained from each subject.

### 2.4. Cultures

To avoid contamination, all cultures were performed under strict aseptic conditions in a laboratory that was free of *Borrelia* reference strains, and cultures of control and patient samples were processed in an identical manner. Inocula were placed in Barbour-Stoner-Kelly H (BSK) complete medium with 6% rabbit serum (Sigma-Aldrich, #B8291, St. Louis, MO, USA) containing the following antibiotics: phosphomycin (0.02 mg/mL) (Sigma-Aldrich), rifampicin (0.05 mg/mL) (Sigma-Aldrich), and amphotericin B (2.5 μg/mL) (Sigma-Aldrich), as described previously [62]. Inocula were prepared as follows: 

A. Blood—whole blood (10 mL) was collected by venipuncture and left at room temperature to clot, then centrifuged at low speed to separate red blood cells from sera. The serum supernatants with a small amount of blood cells below the serum layer were collected and were inoculated into the BSK medium.

B. Skin—whole calluses or skin from lesions were removed from MD subjects by scraping with a scalpel blade.

C. Vaginal—vaginal secretions were collected by swabbing inside the vagina with sterile cotton-tipped swabs that were then introduced into the BSK medium. 

D. Seminal—semen was self-collected into a sterile vial, then was pipetted into the BSK medium.

8 mL tubes of inoculated medium were filled to minimize the airspace present, thus providing a microaerobic environment, and incubated at 32 °C. Culture fluid was examined by darkfield microscopy for visible spirochetes weekly for up to 4 weeks. Cultures were concentrated by centrifuging the fluid at 15,000 *g* for 20 min, retaining the pellet and discarding the supernatant. For imaging, a small amount of culture pellet was resuspended in 50 μL 0.85% saline solution, washed and centrifuged again. The pellet was mixed with gelatin and then fixed with formalin for further staining.

### 2.5. Dieterle and Anti-Bb Immunostaining

Dermatological specimens and/or culture pellets from patients were fixed, sectioned and processed for specialized staining at either McClain Laboratories LLC, Smithtown, NY, USA, or the Department of Biology and Environmental Science, University of New Haven, West Haven, CT, USA, as previously described [59]. Dieterle silver-nitrate staining was performed at McClain Laboratories. Anti-Bb immunostaining was performed at McClain Laboratories or the University of New Haven. In brief, immunostaining was performed using an unconjugated rabbit anti-Bb polyclonal antibody (Abcam ab20950, Cambridge, MA, USA), incubated with an alkaline phosphatase probe (Biocare Medical #UP536L, Pacheco, CA, USA), followed by a chromogen substrate (Biocare Medical #FR805CHC), and counterstained with hematoxylin. Positive and negative controls were prepared for comparison purposes using liver sections from Bb-inoculated and uninfected C3H/HeJ mice followed by Dieterle and immunostaining. Culture pellets from mixed Gram-positive bacteria (*Streptococcus* and *Staphylococcus*) and Gram-negative bacteria (*Escherichia coli* and *Klebsiella*) were also prepared for comparison purposes as negative controls to exclude cross-reactivity with commonly encountered microorganisms.

### 2.6. Molecular Testing

Patient and negative control samples were submitted in a blinded manner to the laboratories performing polymerase chain reaction (PCR) amplification of DNA, as described below. PCR detection of *Borrelia* was performed for research purposes only. No data resulting from this study was used diagnostically.

### 2.7. PCR—University of New Haven 

DNA was extracted from culture pellets as previously described [59]. Reactions of blinded samples were performed in triplicate.

*Borrelia* DNA in extracted samples was detected using a published TaqMan assay targeting a 139-bp fragment of the gene encoding the *Borrelia* 16S rRNA, as described previously [59,63,64]. Amplifications were conducted on a CFX96 Real-Time System (Bio-Rad, Hercules, CA, USA) with cycling of 50 °C for 2 min, 95 °C for 10 min, followed by 40 cycles of 95 °C for 15 s and 60 °C for 60 s, and fluorescent signals were recorded using CFX96 Real-Time software with the Cq threshold set automatically. 

Nested PCR primers for the 16S rRNA, flagellin (Fla), OspC, uvrA and pyrG genes were used as previously described [59,64,65,66], with a final volume of 50 μL using 10 μL template DNA and final concentrations of 20 mM Tris-HCl (pH 8.4), 50 mM KCl (1 × Buffer B, Promega, Fitchburg, WI, USA), 2 mM MgCl_2_, 0.4 mM dNTP mix, 2 μM of each primer, and 2.5 U Taq polymerase (Invitrogen, Carlsbad, CA, USA). The first reaction used “outer” primers and the second reaction used “inner” primers, and 1 μL of PCR product from the first reaction was used as template for the second. Cycling was programmed as follows: 94 °C for 5 min followed by 40 cycles of denaturation at 94 °C for 1 min, annealing for 1 min, and extension at 72 °C for 1 min, with a final extension step at 72 °C for 5 min. DNA products were visualized in 1–2% agarose gels.

PCR amplification was followed by Sanger sequencing. PCR products were extracted using the QIAquick Gel Extraction kit (Qiagen, Hilden, Germany) in accordance with the manufacturer’s instructions. Eluates were sequenced in both directions, then were compared by BLAST analysis using the GenBank database (National Center for Biotechnology Information).

### 2.8. PCR—Australian Biologics

DNA was extracted from culture pellets using the DNeasy Blood and Tissue kit^®^ (Qiagen) in accordance with the manufacturer’s instructions. Samples were forwarded to Australian Biologics for *Borrelia* DNA and *Treponema denticola*/*Treponema pallidum* DNA testing. Blinded samples were run in duplicate with positive and negative controls using primers for the *Borrelia* 16S rRNA and rpoC gene targets, as previously described [59,67,68]. *Borrelia* DNA was detected by real-time PCR targeting the 16S rRNA gene and/or by endpoint PCR targeting the rpoC gene, as previously described [59,67,68], using the Eco™ Real-Time PCR system with software version 3.0.16.0. Thermal profiles were performed with incubation for 2 min at 50 °C, polymerase activation for 10 min at 95 °C then PCR cycling for 40 cycles of 10 s at 95 °C dropping to 60 °C sustained for 45 s. The PCR signal magnitude generated (∆R) was interpreted as either positive or negative as compared to positive and negative controls.

For endpoint PCR, amplicons were visualized on 1–2% agarose gels and extracted from the gels using the QIAquick Gel Extraction kit (Qiagen) in accordance with the manufacturer’s instructions. Sanger sequencing was used for gene analysis, as described previously [59,67,68].

### 2.9. PCR—University California Irvine 

The presence of Bb sensu stricto DNA in a set of blinded samples was confirmed by the laboratory of Dr. Alan Barbour (University of California Irvine) by first quantitative PCR [69], and then by sequence of the PCR-amplified 16S-23S intergenic spacer [70]. The samples studied included specimens from two of the subjects in this paper, Case 2 and Case 10, as described below.

## 3. Results

### 3.1. Subject Histories

The clinical histories of the 12 study subjects with persistent Lyme disease symptoms (Cases 1–12) are provided below, and the clinical characteristics of the subjects are summarized in Table 1. All subjects had received treatment with 2–4 weeks of antibiotics as recommended by Lyme treatment guidelines endorsed by the Centers for Disease Control and Prevention (CDC) [14]. Six patients were taking antibiotics at the time of study sampling, as noted in Table 1. The type of Lyme IgM and IgG Western blot reactivity detected in each patient is shown in Table 2. Persistent IgM reactivity was found in several patients, as described in other studies [37].

Control samples were obtained from two men and eight women ranging in age from 43–63 years (mean age, 50.6 years). None of the controls had symptoms of tickborne disease, and none had received antibiotic therapy. All controls were negative on Lyme IgM and IgG Western blot testing, and additional testing of controls using microscopy, histopathology and PCR techniques was negative, as outlined below.

#### 3.1.1. Case 1

The subject is a 50-year-old native Canadian woman who resided in an area endemic for LD in eastern Canada. She did not recall an erythema migrans (EM) rash. She developed extreme fatigue and musculoskeletal pain as well as ulcerative skin lesions along with symptoms of formication. Magnification demonstrated filamentous inclusions within the lesions. The subject was seronegative for anti-Bb antibodies excepting two indeterminate IgM bands showing reactivity to the 41-kDa and the 93-kDA proteins, and a weakly positive IgG band showing reactivity to the 41-kDa protein. She was clinically diagnosed with LD by a health care provider in Canada and treated with antibiotics. The subject had discontinued antibiotics three weeks prior to the sampling period, but continued treatment with naturopathic remedies. Despite ongoing treatment with amoxicillin, the subject continues to have persistent symptoms of Lyme disease.

#### 3.1.2. Case 2

The subject is a 54-year-old Caucasian woman who had a history of outdoor recreational activity in Western Canada including areas in British Columbia that are endemic for LD. She recalled an EM-like rash several years previously, and she did not receive treatment. She developed significant joint pains, muscle aches, headaches, memory loss, fatigue and skin lesions, and she initially tested negative for Lyme disease. She was clinically diagnosed by a Canadian health care provider, and the diagnosis was confirmed later by an American health care provider. She also had positive serological tests for *Babesia* and *Bartonella*. She did not have prior knowledge of Morgellons disease, but she did have ulcerative lesions on her face and torso consistent in appearance with the condition. Upon examination with a 50× handheld microscope, filamentous inclusions were observed in her lesions. She has been aggressively treated over the last few years with antibiotic combinations including intravenous ceftriaxone, metronidazole, telithromycin, doxycycline, amoxicillin, ciprofloxacin, tinidazole and atovaquone with little benefit.

#### 3.1.3. Case 3

The subject is a 63-year-old Caucasian man who had a history of outdoor recreational activity in endemic areas for Lyme disease, including Europe, Western Canada, and the USA (Connecticut and Rhode Island). Although he recalls tick bites, he did not recall an EM rash. The subject developed musculoskeletal pain and extreme fatigue. His wife (Case 4) had an EM rash and a LD diagnosis that prompted him to get tested for LD. He was seroreactive for anti-Bb antibodies, and Bb DNA was detected in serum using PCR technology. He tested serologically positive for *Babesia microti* and *Anaplasma phagocytophylum*. He had received ongoing treatment with antibiotics, including doxycycline, clarithromycin, cefdinir, tinidazole, atovaquone, clindamycin and hydroxychloroquine. He was symptomatic and taking doxycycline at the time of sampling. His condition has since improved, but he still suffers from musculoskeletal pain.

#### 3.1.4. Case 4

The subject is a 53-year-old Caucasian woman and the wife of Case 3. She had a history of outdoor recreational activity in Lyme endemic areas of the USA and Canada. She has a history of tick bites and recalled an EM rash after visiting both Connecticut and Rhode Island. Her symptoms included seizures, neuropathy, palpitations and musculoskeletal pain. She had serological testing for Bb and was initially negative, but she became seropositive after taking antibiotics. She also had positive serological testing for *Babesia microti* and *Anaplasma phagocytophylum*. She was symptomatic and taking antibiotics during the time of sample collection. Antibiotics taken included doxycycline, telithromycin, minocycline, clindamycin, clarithromycin, metronidazole, tinidazole, rifampicin, atovaquone, hydroxychloroquine and mefloquine. The subject was taking clarithromycin and cefdinir at the time of sample collection. She is currently asymptomatic.

#### 3.1.5. Case 5

The subject is a 40-year-old Caucasian woman living in Calgary, Canada, and the partner of Case 6. She is a veterinarian and had a history of work exposure to ticks, and she had also travelled to areas endemic for LD in Europe. She did not recall an EM rash. Her symptoms were primarily musculoskeletal and severe headaches. She was seropositive for Bb and *Babesia*, and she had been treated with the following antibiotics: doxycycline, clarithromycin, metronidazole and atovaquone. She had been taking doxycycline for one month at the time of sample collection.

#### 3.1.6. Case 6

The subject is a 42-year-old Caucasian man living in Calgary, Canada, and the partner of Case 5. He is a veterinarian and had a history of work exposure to ticks. He had also travelled to areas endemic for LD in Europe. He did not recall an EM rash. His symptoms were primarily musculoskeletal, severe headaches, memory loss, vision problems and extreme fatigue. He was seropositive for Bb and *Babesia*, and he had been treated with the following antibiotics: doxycycline, clarithromycin, metronidazole and atovaquone. He had been taking doxycycline for one month at the time of sample collection.

#### 3.1.7. Case 7

The subject is a 36-year-old Caucasian woman living in Calgary, Canada. She was bitten by many ticks while working as a tree planter in the mountains, but she does not recall an EM rash. In September 1997 she developed profound fatigue, migratory joint pains, peripheral neuropathy and personality changes consistent with depression. She was seropositive for Bb, and she was eventually treated with intramuscular penicillin, amoxicillin, and minocycline over two years. She remains symptomatic despite antibiotic treatment.

#### 3.1.8. Case 8

The subject is a 39-year-old Caucasian man residing in Calgary, Canada. He has a history of hiking, camping and other outdoor activities in Alberta and Manitoba, Canada, but no known tick bites or EM rash. He complains of joint pain, low back pain and headaches, and he has been treated for sciatica, depression, insomnia, and anxiety. He also has an extensive history of periodontal disease with recurrent gingival infections, and he has received multiple courses of penicillin and amoxicillin over many years. He had positive serological testing for Lyme disease, and he has not been tested for tickborne coinfections. 

#### 3.1.9. Case 9

The subject is a 71-year-old Caucasian woman living in Ontario, Canada and the partner of Case 10. She was 40 years old when she became ill in 1986 with severe flu-like symptoms, fatigue, severe pelvic pain, blurred vision, rib soreness and night sweats. She did not recall a tick bite or an EM rash. The patient had not knowingly visited a Lyme disease endemic area. She consulted six different physicians over a period of four years before being treated with six weeks of doxycycline for what was diagnosed as pelvic inflammatory disease in 1988, and her symptoms transiently improved. She was clinically diagnosed with Lyme disease in 1990 by a physician in Ontario, as the Ontario government’s ELISA test was “negative” for Lyme disease. Over the next 20 years the subject was intermittently treated with doxycycline and her symptoms improved, but never completely resolved, and other symptoms developed such as muscle aches, joint pains, sleep disturbances, bladder and urethral pain, and cognitive impairment. These symptoms waxed and waned over the years. She experienced multiple Jarisch–Herxheimer reactions with repeated doxycycline treatment. The subject’s two children were treated for congenital Lyme disease between 1990 and 2004 and are asymptomatic today. In May 2011, the subject was tested by a CLIA-approved laboratory in the USA and was found to be serologically positive for Lyme disease. 

#### 3.1.10. Case 10

The subject is a 72-year-old Caucasian man living in Ontario, Canada and the partner of Case 9. He was 41 years old at the onset of symptoms in 1986 with flu-like muscle aches, joint pains and unrelenting fatigue. He did not recall a tick bite or an EM rash. The subject had not knowingly visited a Lyme disease endemic area. He had consulted 12 different doctors over a period of four years before getting a confirmed diagnosis of Lyme disease, at which point he had developed severe arthritic symptoms, significant neurological symptoms including encephalopathy and dementia with brain magnetic resonance imaging (MRI) showing hyperintense white matter lesions. His antibiotic regimens over 20 years included: tetracycline, amoxicillin plus probenecid, doxycycline, clarithromycin, intravenous ceftriaxone, and intramuscular benzathine penicillin G. When the subject was on antibiotics he had relief of many symptoms, but he was never completely free of symptoms associated with Lyme disease. He had multiple Jarisch-Herxheimer reactions when new antibiotic regimens were initiated. The two-tiered Lyme disease serology test performed in Canada failed to show positivity for Lyme disease, but the subject subsequently sent blood to a CLIA-approved laboratory in the United States and was found to be seropositive for Lyme disease.

#### 3.1.11. Case 11

The subject is a 57-year-old Caucasian woman living in Calgary, Canada. She had exposure to ticks while hiking and camping in Canada, but she did not recall an EM rash. She developed musculoskeletal and neuropsychiatric symptoms and was diagnosed with LD after testing serologically positive. She also had positive testing for *Babesia, Ehrlichia* and *Bartonella*. She received intermittent antibiotic therapy with multiple oral, intramuscular and intravenous antibiotics, and her symptoms improved while on antibiotics but relapsed when the antibiotics were discontinued. She remains symptomatic after five years of antibiotic treatment.

#### 3.1.12. Case 12

The subject is a 46-year-old Caucasian woman living in Alberta, Canada who did not have a history of tick bite or EM rash. She has suffered with skin lesions consistent with Morgellons disease for more than a decade and was diagnosed with Lyme disease in the USA after testing serologically positive for Bb. She has had severe gastrointestinal problems that have necessitated frequent hospitalizations. The gastrointestinal difficulties began after she had gastric bypass surgery. Her intestines form lesions that fuse together, causing blockages. She has been treated aggressively with antibiotics before and after this study by doctors both in the USA and Canada with only minimal benefit. Antibiotic therapy included multiple treatments with intravenous ceftriaxone, doxycycline, clarithromycin and amoxicillin.

### 3.2. Microscopy and Histopathology

Case 1. Whole calluses were submitted for sectioning, Dieterle and anti-Bb immunostaining. Spirochetes were visible in both Dieterle and anti-Bb immunostains. Blood culture was performed and fluid from the culture demonstrated spherical bodies under darkfield microscopy. Dieterle staining and anti-Bb immunostaining was not performed.

Case 2. Blood culture was performed and fluid from the culture demonstrated spherical bodies under darkfield microscopy. Dieterle staining and anti-Bb immunostains demonstrated spherical bodies. Anti-Bb immunostaining was positive. Vaginal culture was performed and fluid from the culture demonstrated spirochetes and biofilm under darkfield microscopy. Dieterle staining and anti-Bb immunostaining demonstrated spirochetes and biofilm. Skin specimens were not submitted for staining or culture. Repeat blood and vaginal cultures were positive for *Borrelia* by immunostaining and PCR (see Table 3, Table 4 and Table 5).

Case 3. Blood culture was performed and fluid from the culture demonstrated spherical bodies and occasional spirochetes under darkfield microscopy. Dieterle staining and anti-Bb immunostains demonstrated spherical bodies and occasional spirochetes. Anti-Bb immunostaining was positive. Seminal culture was performed and fluid from the culture demonstrated spirochetes under darkfield microscopy. Dieterle staining and anti-Bb immunostaining demonstrated spirochetes. Repeat blood culture was positive for *Borrelia* by immunostaining and PCR (see Table 3, Table 4 and Table 5).

Case 4. Blood culture was performed and fluid from the culture demonstrated spherical bodies and occasional spirochetes under darkfield microscopy. Dieterle staining and anti-Bb immunostains demonstrated spherical bodies and occasional spirochetes. Anti-Bb immunostaining was positive. Vaginal culture was performed and fluid from the culture demonstrated spirochetes under darkfield microscopy. Dieterle staining and anti-Bb immunostaining demonstrated spirochetes. Repeat blood culture was positive for *Borrelia* by immunostaining and PCR (see Table 3, Table 4 and Table 5).

Case 5. Blood culture was performed and fluid from the culture demonstrated spherical bodies under darkfield microscopy. Dieterle staining and anti-Bb immunostaining demonstrated spherical bodies. Vaginal culture was performed and fluid from the culture demonstrated spirochetes under darkfield microscopy. Dieterle staining and anti-Bb immunostaining demonstrated spirochetes. Repeat blood culture was positive for *Borrelia* by immunostaining and PCR (see Table 3, Table 4 and Table 5).

Case 6. Blood culture was performed and fluid from the culture demonstrated spherical bodies under darkfield microscopy. Dieterle staining and anti-Bb immunostains demonstrated spherical bodies. Anti-Bb immunostaining was positive. Seminal culture was performed and fluid from the culture demonstrated spirochetes under darkfield microscopy. Dieterle staining and anti-Bb immunostaining demonstrated spirochetes. Repeat blood culture was positive for *Borrelia* by immunostaining and PCR (see Table 3, Table 4 and Table 5).

Case 7. Vaginal culture was performed and fluid from the culture demonstrated spirochetes under darkfield microscopy. Dieterle staining and anti-Bb immunostaining demonstrated spirochetes.

Case 8. Seminal culture was performed and fluid from the culture demonstrated spirochetes under darkfield microscopy. Dieterle staining and anti-Bb immunostaining demonstrated spirochetes.

Case 9. Vaginal culture was performed and fluid from the culture demonstrated spirochetes, including one that was quite actively motile, under darkfield microscopy. Dieterle staining and anti-Bb immunostaining demonstrated spirochetes. 

Case 10. Seminal culture was performed and fluid from the culture demonstrated spirochetes under darkfield microscopy. Dieterle staining and anti-Bb immunostaining demonstrated spirochetes. Repeat seminal culture was positive for *Borrelia* by immunostaining and PCR (see Table 3, Table 4 and Table 5).

Case 11. Vaginal culture was performed and fluid from the culture demonstrated spirochetes, including one that was quite actively motile, under darkfield microscopy. Dieterle staining and anti-Bb immunostaining demonstrated spirochetes.

Case 12. Blood culture was performed and fluid from the culture demonstrated spherical bodies under darkfield microscopy. Dieterle staining and anti-Bb immunostains demonstrated spherical bodies. Anti-Bb immunostaining was positive. Skin culture was performed and fluid from the culture demonstrated spirochetes under darkfield microscopy. Dieterle staining and anti-Bb immunostaining demonstrated spirochetes.

**Figure 1:**
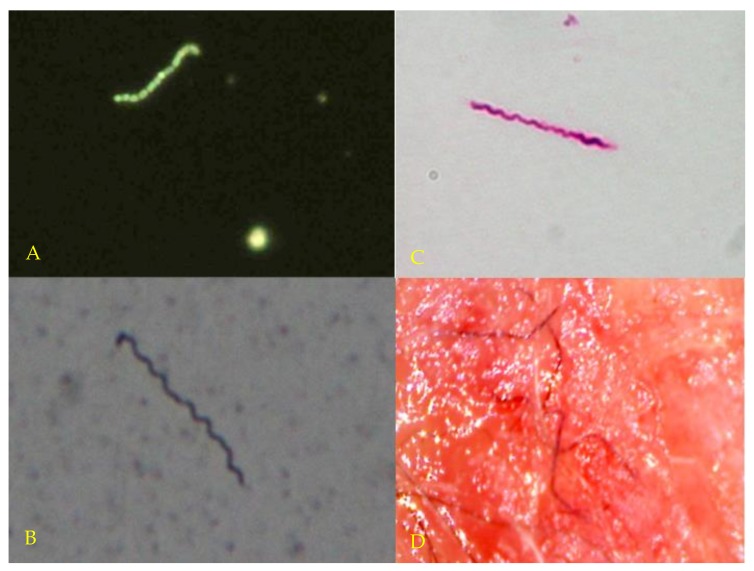
(**A**) (Top left): Darkfield microscopy of blood culture showing live spirochete and spherules. Magnification 400×. (**B**) (Bottom left): Dieterle silver stain of culture fluid from Case 10 showing live spirochetes. Magnification 1000×. (**C**) (Top right): *Borrelia* immunostain of culture fluid from Case 9 showing live spirochetes. Magnification 1000×. (**D**) (Bottom right). Typical dermal filaments from patient with Morgellons disease. Magnification 100×.

The results of darkfield microscopy, Dieterle silver stains and anti-Bb immunostaining from Cases 1–12 are summarized in Table 3. Examples of these spirochete detection methods are shown in Figure 1A–C. All controls tested negative using these techniques (data not shown).

### 3.3. Molecular Testing

#### PCR Detection of *Borrelia*

Samples (whole dermatological calluses, blood culture, vaginal cultures, and seminal cultures) from the study patients were submitted for PCR detection and sequencing of *Borrelia* DNA at both the University of New Haven, CT, and Australian Biologics, Sydney, Australia in a blinded manner. *Borrelia* DNA was detected by at least one laboratory for all 12 patients, and amplicon sequences consistent with Bb DNA were obtained for 10/12 patients. Blinded negative cultures from healthy, seronegative subjects along with the blinded suspected positive cultures from LD patients were sent to University of New Haven and Australian Biologics.

Australian Biologics performed PCR for the detection of *Treponema denticola* and *Treponema pallidum* on all study samples and blinded controls. Treponemal DNA was not detected in any samples. For Cases #2 and #10, additional PCR and sequencing on genital cultures was performed at the University of California, Irvine, in the laboratory of Dr. Alan Barbour. In these samples, *Borrelia* DNA was detected with qPCR and then confirmed as Bb sensu stricto by sequence of the 16S-23S intergenic spacer.

Positive PCR results are summarized in Table 4, and positive sequencing results are summarized in Table 5. PCR sequences and BLAST analyses are shown in Supplemental File. All negative controls were PCR-negative for *Borrelia* species (data not shown).

## 4. Discussion

In this pilot study, we cultured live *Borrelia* organisms from 12 antibiotic-treated subjects with persistent Lyme disease symptoms, thus showing that viable spirochetes can be found in LD patients despite antibiotic therapy. Half of these subjects were taking antibiotics at the time of sampling. Patient cultures showed *Borrelia* spiral forms and spherical bodies, as described in other publications (Figure 1A–C) [7,59,66]. We demonstrated the presence of *Borrelia* infection in cultures from these patients using corroborative microscopy, histopathology and PCR techniques, and we obtained sequences for amplicons from 10/12 patients. Repeat cultures of blood, semen and vaginal secretions were positive for Bb by microscopy, histopathology and PCR in six patients tested by four different laboratories. Cultures from healthy *Borrelia*-seronegative controls were consistently negative using microscopy, histopathology and PCR techniques, making the possibility of *Borrelia* contamination in LD patient samples extremely unlikely.

Persistent *Borrelia* infection may result in part from the wide variety of tissues and fluids that support spirochetal growth [16,17,18,19,20,21,22,23,24,25,26,27,28,29,30,31,32,33,34,35,36,37]. The tissues susceptible to *Borrelia* infection include fibroblasts, skin, synovial tissue, ligaments, cardiac tissue, glial cells, neurons, endothelial cells, lymphoid tissue and hepatic tissue [5,48,59,71,72,73,74,75,76,77,78,79,80,81]. The pleotropic nature of *Borrelia* infection may allow the spirochete to evade the host immune system and antibiotic therapy, as outlined below.

The role of round body cysts and biofilms in persistent *Borrelia* infection is controversial [10,82,83]. Ongoing Lyme disease symptoms may arise from spirochetes hidden in biofilms or surviving as round body cysts or cell wall-deficient L-forms, by intracellular *Borrelia* sequestration or by sequestration within privileged sites where antibiotics do not attain therapeutic levels [13,37,84,85,86]. Regardless of the mechanism by which *Borrelia* spirochetes persist in tissues, persistent *Borrelia* infection requires treatment, and options at present are limited and controversial [10,13,83]. The controversy is fueled by disagreement over viability of the spirochetes, as described below.

Although there is evidence of post-treatment *Borrelia* infection in animals and humans, some researchers speculate that *Borrelia* antigens and DNA detected in studies are merely spirochetal “debris” [36,87,88,89]. Wormser et al. offered an “amber” hypothesis as a possible explanation for persistent symptoms, namely that persistent Lyme arthritis is caused by non-viable spirochetes enmeshed in joints within host-derived fibrinous or collagenous matrices [88]. Bockenstedt et al. proposed that the inflammation seen in mice described in their study following antibiotic treatment was caused by *Borrelia* DNA and proteins representing non-infectious spirochetal “debris” deposited in tissues [36].

In contrast, those who support the idea that active infection is responsible for persisting Lyme disease symptoms propose that there are various protective mechanisms providing spirochetal resistance or tolerance to antibiotics, including intracellular invasion and formation of cell-wall deficient L-forms, round body cysts, biofilms and persister cells [78,84,85,86]. Furthermore the “amber” and “debris” hypotheses of symptom persistence are difficult to support because *Borrelia* DNA is rapidly cleared from murine tissues after prompt antibiotic treatment [21], and the DNA of non-viable spirochetes is cleared from mouse tissue within several hours [90]. The present study confirms the presence of live *Borrelia* spirochetes in patients who had been treated with antibiotics for persistent Lyme disease symptoms.

Recent studies have focused on “persister cells” and “sleeper cells” as spirochetal agents of persistence in Lyme disease [91,92,93]. The concept involves organisms that are tolerant to antibiotics and can downregulate their metabolic needs via a “stringent response” to survive in a hostile environment, only to reemerge when the environment becomes more favorable. A similar mechanism of persistent infection has been described in *E. coli*, *Mycobacteria* and *Salmonella* [93]. The survival of metabolically tolerant spirochetes in privileged sites would explain our findings of viable *Borrelia* in antibiotic-treated patients once the antibiotics are withdrawn and culture conditions are optimized. The factors that influence viability of “persister cells” and “sleeper cells” in patients with persistent Lyme disease symptoms merit further study.

Three of our study subjects had a controversial skin condition commonly called Morgellons disease (MD) [61,94,95,96,97,98]. The distinguishing feature of this skin condition is the presence of white, black, or brightly colored filaments that lie under, are embedded in, or project from skin lesions (see Figure 1D). While some medical practitioners erroneously consider MD to be a purely delusional disorder, MD appears to be a *Borrelia*-associated filamentous dermatitis [94,95]. MD patients exhibit symptoms that resemble those of Lyme disease such as fatigue, joint pain, and neuropathy, and the skin condition has been shown to be associated with *Borrelia* infection [94,95,96,97,98]. Spirochetes from different *Borrelia* species have been detected in MD patient specimens [61,94,99,100]. We obtained positive *Borrelia* cultures from all three of our MD subjects.

The mechanism of MD filament evolution has not been resolved, but as collagen and keratin filaments arise from proliferative keratinocytes and fibroblasts in human epithelial tissue, we speculate that *Borrelia* infection alters keratin and collagen gene regulation [99,100]. *Borrelia* bacteria can invade fibroblasts and keratinocytes where they survive and replicate intracellularly [74,76,101]. As shown by in vitro studies, *Borrelia* spirochetes can be isolated from keratinocyte and fibroblast monolayers despite treatment with antibiotics [74,76]. Persistent refractory infection in MD patients may therefore result in part from sequestration of live *Borrelia* spirochetes within keratinocytes and fibroblasts.

*Borrelia* spirochetes have been detected in vaginal and seminal secretions [13,100]. We cultured *Borrelia* spirochetes in genital secretions from ten of our study subjects who had taken or were currently taking antibiotic therapy. Bb is a complex organism that is related to the spirochetal agent of syphilis, and therefore may have similar infectious capabilities [13,100,102]. As outlined above, *Borrelia* spirochetes penetrate tissues, can form cystic structures and L-forms, hide in biofilms, become intracellular, and sequester in privileged sites (brain, eye and synovium) [9,10,13,83,103,104,105]. These specialized abilities of the *Borrelia* spirochete suggest that the genital tract could harbor infection. The vagina and the seminal vesicles are privileged sites, and that may explain why the organism can persist in the genital tract despite antimicrobial therapy in a manner similar to syphilis, chlamydia, human immunodeficiency virus (HIV), Ebola and Zika virus [102,106,107,108,109,110].

## 5. Conclusions

In summary, in this pilot study we demonstrated persistent infection despite antibiotic therapy in 12 North American patients with ongoing symptoms of LD. Cultures were positive in all 12 patients in our study, indicating that the *Borrelia* spirochetes were replicating and therefore alive. The spirochetes were genetically identified as Bb in a blinded fashion using PCR and gene sequencing in three separate laboratories. In contrast, cultures from control subjects without Lyme disease were negative for *Borrelia* spirochetes. Our findings provide evidence that persistent infection rather than spirochetal “debris” was at least in part responsible for ongoing symptoms in these cases of Lyme disease, and the results mirror recent observations in a non-human primate model of treated Lyme disease [37]. Larger clinical studies using corroborative techniques are needed to confirm the findings in this pilot study.

## Figures and Tables

**Table 1:** Clinical Characteristics of Study Patients.

Case #	Age/Gender	EM Rash	Sx	MD Lesions	LD Seroreactivity	Co-Infections	Abx
Case 1	50F	No	MS, F	Yes	Negative	Unknown	Yes
Case 2	54F	Yes	MS, F	Yes	Negative	*Bab*, *Bart*	No
Case 3	63M	No	MS, F	No	Positive	*Bab*, *Ana*	Yes
Case 4	53F	Yes	MS, F	No	Negative, seroconverting to positive	*Bab*, *Ana*	Yes
Case 5	40F	No	MS, N	No	Positive	*Bab*	Yes
Case 6	42M	No	MS, N	No	Positive	None	Yes
Case 7	36F	No	MS, N	No	Positive	None	Yes
Case 8	39M	No	MS, N	No	Positive	Unknown	No
Case 9	71F	No	MS, F, N	No	Positive	None	No
Case 10	72M	No	MS, F, N	No	Positive	None	No
Case 11	57F	No	MS	No	Positive	*Bab*, *Ehr*, *Bart*	No
Case 12	46F	No	MS	Yes	Positive	*Bab*	No

EM, erythema migrans; MD, Morgellons disease; MS, musculoskeletal; F, fatigue; N, neurological; *Bab*, *Babesia microti* or *Babesia duncani*; *Bart*, *Bartonella henselae; Ana*, *Anaplasma phagocytophilum*; *Ehr*, *Ehrlichia chafeensis.* Abx, on antibiotics at time of testing.

**Table 2:** Lyme Western Blot IgM and IgG Results in Study Patients.

Patient Number	Western Blot IgM	Western Blot IgG
1	Negative	Negative
2	Negative	Negative
3	Negative	Positive
4	Positive	Negative
5	Positive	Negative
6	Positive	Negative
7	Positive	Negative
8	Positive	Negative
9	Positive	Positive
10	Positive	Negative
11	Positive	Positive
12	Negative	Positive

Western blots were interpreted according to IGeneX criteria [60].

**Table 3:** Summary of Microscopy Results from Patient Culture Samples.

Case #	Sample Type	Darkfield	Dieterle	Bb Immunostain
Case 1	whole callus	N/A	spirochetes	positive, spirochetes
	blood culture	spirochetes	N/A	N/A
Case 2	blood culture	spherules	spherules	positive, spherules
	vaginal culture	spirochetes	spirochetes	positive, spirochetes, biofilm
Case 3	blood culture	spirochetes/spherules	spirochetes/spherules	positive spirochetes/spherules
	seminal culture	spirochetes	spirochetes	positive, spirochetes
Case 4	blood culture	spirochetes/spherules	spirochetes/spherules	positive spirochetes/spherules
	vaginal culture	spirochetes	spirochetes	positive, spirochetes
Case 5	blood culture	spherules	spherules	positive, spherules
	vaginal culture	spirochetes	spirochetes	positive, spirochetes
Case 6	blood culture	spherules	spherules	positive, spherules
	seminal culture	spirochetes	spirochetes	positive, spirochetes
Case 7	vaginal culture	spirochetes	spirochetes	positive, spirochetes
Case 8	seminal culture	spirochetes	spirochetes	positive, spirochetes
Case 9	vaginal culture	spirochetes	spirochetes	positive, spirochetes
Case 10	seminal culture	spirochetes	spirochetes	positive, spirochetes
Case 11	vaginal culture	spirochetes	spirochetes	positive, spirochetes
Case 12	blood culture	spherules	spherules	positive, spherules
	skin culture	spirochetes	spirochetes	positive, spirochetes

N/A, not available.

**Table 4:** Summary of PCR Results from Patient Culture Samples.

Case #	Sample Type	University of New Haven	Australian Biologics	UC-Irvine
1	whole callus	16S rRNA (N), pyrG (N) *, fla (N) *	N/A	N/A
	blood culture	pyrG (N), fla (N) *	N/A	N/A
2	blood culture	16S rRNA (N)	16S rRNA (RT), rpoC (E) *	N/A
	vaginal culture	pyrG (N) *, fla (N)	16S rRNA (RT) *	qPCR 16S-23S intergenic spacer
3	blood culture	16S rRNA (N) *	16S rRNA (RT)	N/A
	seminal culture	16S rRNA (RT), 16S rRNA (N) *, fla (N)	16S rRNA (RT), rpoC (E) *	N/A
4	blood culture	16S rRNA (N), pyrG (N)	16S rRNA (RT), rpoC (E) *	N/A
	vaginal culture	16S rRNA (RT), 16S rRNA (N), pyrG (N), fla (N)	16S rRNA (RT), rpoC (E) *	N/A
5	blood culture	16S rRNA (RT), 16S rRNA (N), pyrG (N)	16S rRNA (RT)	N/A
	vaginal culture	16S rRNA (N)	16S rRNA (RT), rpoC (E) *	N/A
6	blood culture	16S rRNA (RT), 16S rRNA (N), pyrG (N)	16S rRNA (RT)	N/A
	seminal culture	16S rRNA (RT), 16S rRNA (N)	16S rRNA (RT)	N/A
7	vaginal culture	16S rRNA (N) *	N/A	N/A
8	seminal culture	16S rRNA (N) *	N/A	N/A
9	vaginal culture	pyrG (N)	16S rRNA (RT)	N/A
10	seminal culture	pyrG (N)	16S rRNA (RT)	(+/−) qPCR16S-23S intergenic spacer
11	vaginal culture	16S rRNA (N)	16S rRNA (RT), rpoC (E) *	N/A
12	whole callus	uvrA (N) *	N/A	N/A
	blood culture	pyrG	16S rRNA (RT)	N/A

* Sequenced; RT, real time PCR; N, nested PCR; E, endpoint PCR; N/A not available; (+/−) One specimen was positive for Bb DNA, one specimen was negative (different collection dates).

**Table 5:** Summary of BLAST Sequence Analysis of Patient PCR Samples.

Case #	Culture Specimen	Sequence	Length	E-Value	BLAST Match Bbss	LAB
1	callus	pyrG	680	0.0	100%	UNH
	callus	fla	367	2e-172	100%	UNH
	blood	fla F	364	2e-176	99%	UNH
	blood	fla R	367	2e-172	99%	UNH
2	vaginal	pyrG F	656	0.0	99%	UNH
	vaginal	pyrG R	659	0.0	99%	UNH
	vaginal	rpoC	79	4e-32	100%	AB
	vaginal	16S-23S Intergenic spacer	474	0.0	100%	UCI
3	blood	16S rRNA F	415	0.0	99%	UNH
	blood	16S rRNA R	415	0.0	99%	UNH
	seminal	16S rRNA	388	0.0	99%	UNH
	blood 1 month Abx	rpoC	103	0.11	96%	AB
	seminal 1 month Abx	rpoC	146	9e-57	100%	AB
	seminal 4 months Abx	rpoC	158	3e-52	98%	AB
4	vaginal	rpoC	118	1e-51	99%	AB
5	vaginal	rpoC	109	6e-47	99%	AB
7	vaginal	16S rRNA	396	0.0	99%	UNH
8	seminal	16S rRNA	221	7e-10	100%	UNH
10	seminal	16S-23S Intergenic spacer	474	0.0	99%	UCI
11	vaginal	rpoC	156	1e-25	100%	AB
12	callus	uvrA F	653	0.0	99%	UNH
	callus	uvrA R	651	0.0	99%	UNH

UNH, University of New Haven; AB, Australian Biologics; UCI, University of California Irvine. F, forward sequence; R, reverse sequence; Bbss, *B. burgdorferi* sensu stricto; Abx, antibiotics.

## Supplementary Materials

The following are available online at http://www.mdpi.com/2227-9032/6/2/33/s1, Supplemental File: PCR Sequences and BLAST Analyses from Individual Patient Cultures.

## References

[1] Steere A.C., Malawista S.E., Snydman D.R., Shope R.E., Andiman W.A., Ross M.R., Steele F.M. (1977). Lyme arthritis: An epidemic of oligoarticular arthritis in children and adults in three Connecticut Communities. Arthritis Rheum..

[2] Stanek G., Reiter M. (2011). The expanding Lyme Borrelia complex—Clinical significance of genomic species?. Clin. Microbiol. Infect..

[3] Cutler S.J., Ruzic-Sabljic E., Potkonjak A. (2017). Emerging borreliae—Expanding beyond Lyme borreliosis. Mol. Cell. Probes.

[4] Rudenko N., Golovchenko M., Vancova M., Clark K., Grubhoffer L., Oliver J.H. (2016). Isolation of live *Borrelia burgdorferi* sensu lato spirochaetes from patients with undefined disorders and symptoms not typical for Lyme borreliosis. Clin. Microbiol. Infect..

[5] Dorward D.W., Fischer E.R., Brooks D.M. (1997). Invasion and cytopathic killing of human lymphocytes by spirochetes causing Lyme disease. Clin. Infect. Dis..

[6] Cameron D., Johnson L.B., Maloney E.L. (2014). Evidence assessments and guideline recommendations in Lyme disease: The clinical management of known tick bites, erythema migrans rashes and persistent disease. Expert Rev. Anti Infect. Ther..

[7] Meriläinen L., Brander H., Herranen A., Schwarzbach A., Gilbert L. (2016). Pleomorphic forms of *Borrelia burgdorferi* induce distinct immune responses. Microbes Infect..

[8] Miklossy J. (2016). Bacterial amyloid and DNA are important constituents of senile plaques: Further evidence of the spirochetal and biofilm nature of senile plaques. J. Alzheimers Dis..

[9] Stricker R.B., Johnson L. (2010). Lyme disease diagnosis and treatment: Lessons from the AIDS epidemic. Minerva Med..

[10] Stricker R.B., Johnson L. (2011). Lyme disease: The next decade. Infect. Drug Resist..

[11] Caruso V.G. (1985). Facial paralysis from Lyme disease. Otolaryngol. Head Neck Surg..

[12] Habicht G.S., Beck G., Benach J.L. (1987). Lyme disease. Sci. Am..

[13] Stricker R.B., Middelveen M.J. (2015). Sexual transmission of Lyme disease: Challenging the tickborne disease paradigm. Expert Rev. Anti Infect. Ther..

[14] Feder H.M., Johnson B.J.B., O’Connell S., Shapiro E.D., Steere A.C., Wormser G.P. (2007). Ad Hoc International Lyme Disease Group. A critical appraisal of “chronic Lyme disease”. N. Engl. J. Med..

[15] Stricker R.B., Johnson L. (2012). Spirochetal ‘debris’ versus persistent infection in chronic Lyme disease: From semantics to science. Fut. Microbiol..

[16] Duray P.H., Johnson R.C. (1986). The histopathology of experimentally infected hamsters with the Lyme disease spirochete *Borrelia burgdorferi*. Proc. Soc. Exp. Biol. Med..

[17] Moody K.D., Barthold S.W., Terwilliger G.A. (1990). Lyme borreliosis in laboratory animals: Effect of host species and in vitro passage *Borrelia burgdorferi*. Am. J. Trop. Med. Hyg..

[18] Preac Mursic V., Patsouris E., Wilske B., Reinhardt S., Gross B., Mehraein P. (1990). Persistence of *Borrelia burgdorferi* and histopathological alterations in experimentally infected animals. A comparison with histopathological findings in human Lyme disease. Infection.

[19] Goodman J.L., Jurkovich P., Kodner C., Johnson R.C. (1991). Persistent cardiac and urinary tract infections with *Borrelia burgdorferi* in experimentally infected Syrian hamsters. J. Clin. Microbiol..

[20] Schmitz J.L., Schell R.F., Lovrich S.D., Callister S.M., Coe J.E. (1991). Characterization of the protective antibody response to *Borrelia burgdorferi* in experimentally infected LSH hamsters. Infect. Immun..

[21] Malawista S.E., Barthold S.W., Persing D.H. (1994). Fate of *Borrelia burgdorferi* DNA in tissues of infected mice after antibiotic treatment. J. Infect. Dis..

[22] Moody K.D., Adams R.L., Barthold S.W. (1994). Effectiveness of antimicrobial treatment against *Borrelia burgdorferi* infection in mice. Antimicrob. Agents Chemother..

[23] Sonnesyn S.W., Manivel J.C., Johnson R.C., Goodman J.L. (1993). A guinea pig model for Lyme disease. Infect. Immun..

[24] Roberts E.D., Bohm R.P., Cogswell F.B., Lanners H.N., Lowrie R.C., Povinelli L., Piesman J., Philipp M.T. (1995). Chronic Lyme disease in the rhesus monkey. Lab. Invest..

[25] Straubinger R.K., Summers B.A., Chang Y.F., Appel M.J. (1997). Persistence of *Borrelia burgdorferi* in experimentally infected dogs after antibiotic treatment. J. Clin. Microbiol..

[26] Straubinger R.K. (2000). PCR-Based quantification of *Borrelia burgdorferi* organisms in canine tissues over a 500-Day postinfection period. J. Clin. Microbiol..

[27] Pachner A.R., Cadavid D., Shu G., Dail D., Pachner S., Hodzic E., Barthold S.W. (2001). Central and peripheral nervous system infection, immunity, and inflammation in the NHP model of Lyme borreliosis. Ann. Neurol..

[28] Cadavid D., Bai Y., Hodzic E., Narayan K., Barthold S.W., Pachner A.R. (2004). Cardiac involvement in non-human primates infected with the Lyme disease spirochete *Borrelia burgdorferi*. Lab. Invest..

[29] Chang Y.F., Ku Y.W., Chang C.F., Chang C.D., McDonough S.P., Divers T., Pough M., Torres A. (2005). Antibiotic treatment of experimentally *Borrelia burgdorferi*-infected ponies. Vet. Microbiol..

[30] Miller J.C., Narayan K., Stevenson B., Pachner A.R. (2005). Expression of *Borrelia burgdorferi* erp genes during infection of non-human primates. Microb. Pathog..

[31] Bockenstedt L.K., Mao J., Hodzic E., Barthold S.W., Fish D. (2002). Detection of attenuated, noninfectious spirochetes in *Borrelia burgdorferi*-infected mice after antibiotic treatment. J. Infect. Dis..

[32] Hodzic E., Feng S., Holden K., Freet K.J., Barthold S.W. (2008). Persistence of *Borrelia burgdorferi* following antibiotic treatment in mice. Antimicrob. Agents Chemother..

[33] Barthold S.W., Hodzic E., Imai D.M., Feng S., Yang X., Luft B.J. (2010). Ineffectiveness of tigecycline against persistent *Borrelia burgdorferi*. Antimicrob. Agents Chemother..

[34] Yrjänäinen H., Hytönen J., Hartiala P., Oski J., Vijanen M.K. (2010). Persistence of borrelial DNA in the joints of *Borrelia burgdorferi*-infected mice after ceftriaxone treatment. APMIS.

[35] Imai D.M., Barr B.C., Daft B., Bertone J.J., Feng S., Hodzic E., Johnston J.M., Olsen K.J., Barthold S.W. (2011). Lyme neuroborreliosis in 2 horses. Vet. Pathol..

[36] Bockenstedt L.K., Gonzales D.G., Haberman A.M., Belperron A.A. (2012). Spirochete antigens persist near cartilage after murine Lyme borreliosis therapy. J. Clin. Investig..

[37] Embers M.E., Hasenkampf N.R., Jacobs M.B., Tardo A.C., Doyle-Meyers L.A., Philipp M.T., Hodzic E. (2017). Variable manifestations, diverse seroreactivity and post-treatment persistence in non-human primates exposed to *Borrelia burgdorferi* by tick feeding. PLoS ONE.

[38] Craft J.E., Fischer D.K., Shimamoto G.T., Steere A.C. (1986). Antigens of *Borrelia burgdorferi* recognized during Lyme disease. Appearance of a new immunoglobulin M response and expansion of the immunoglobulin G response late in the illness. J. Clin. Investig..

[39] Cimmino M.A., Azzolini A., Tobia F., Pesce C.M. (1989). Spirochetes in the spleen of a patient with chronic Lyme disease. Am. J. Clin. Pathol..

[40] De Koning J., Hoogkamp-Korstanje J.A., van der Linde M.R., Crijns H.J. (1989). Demonstration of spirochetes in cardiac biopsies of patients with Lyme disease. J. Infect. Dis..

[41] Preac-Mursic V., Weber K., Pfister H.W., Wilske B., Gross B., Baumann A., Prokop J. (1989). Survival of *Borrelia burgdorferi* in antibiotically treated patients with Lyme borreliosis. Infection.

[42] Fraser D.D., Kong L.I., Miller F.W. (1992). Molecular detection of persistent *Borrelia burgdorferi* in a man with dermatomyositis. Clin. Exp. Rheumatol..

[43] Battafarano D.F., Combs J.A., Enzenauer R.J., Fitxpatrick J.E. (1993). Chronic septic arthritis caused by *Borrelia burgdorferi*. Clin. Orthop. Relat. Res..

[44] Liegner K.B., Shapiro J.R., Ramsay D., Halperin A.J., Hogrefe W., Kong L. (1993). Recurrent erythema migrans despite extended antibiotic treatment with minocycline in a patient with persisting *Borrelia burgdorferi* infection. J. Am. Acad. Dermatol..

[45] Asch E.S., Bujak D.I., Weiss M., Peterson M.G., Weinstein A. (1994). Lyme disease: An infectious and postinfectious syndrome. J. Rheumatol..

[46] Nocton J.J., Dressler F., Rutledge B.J., Rys P.N., Persing D.H., Steere A.C. (1994). Detection of *Borrelia burgdorferi* DNA by polymerase chain reaction in synovial fluid from patients with Lyme arthritis. N. Engl. J. Med..

[47] Bayer M.E., Zhang L., Bayer M.H. (1996). *Borrelia burgdorferi* DNA in the urine of treated patients with chronic Lyme disease symptoms. A PCR study of 97 cases. Infection.

[48] Valesova M., Trnavský K., Hulínská D., Alusík S., Janousek J., Jirous J. (1989). Detection of Borrelia in the synovial tissue from a patient with Lyme borreliosis by electron microscopy. J. Rheumatol..

[49] Donta S.T. (1997). Tetracycline therapy for chronic Lyme disease. Clin. Infect. Dis..

[50] Priem S., Burmester G.R., Kamradt T., Wolbart K., Rittig M.G., Krause A. (1998). Detection of *Borrelia burgdorferi* by polymerase chain reaction in synovial membrane, but not in synovial fluid from patients with persisting Lyme arthritis after antibiotic therapy. Ann. Rheum. Dis..

[51] Hudson B.J., Stewart M., Lennox V.A., Fukunaga M., Yabuki M., Macorison H., Kitchener-Smith J. (1998). Culture-positive Lyme borreliosis. Med. J. Aust..

[52] Oksi J., Nikoskelainen J., Vilajanen M.K. (1998). Comparison of oral cefixime and intravenous ceftriaxone followed by oral amoxicillin in disseminated Lyme borreliosis. Eur. J. Clin. Microbiol. Infect. Dis..

[53] Oksi J., Marjamäki M., Nikoskelainen J., Vilajanen M.K. (1999). *Borrelia burgdorferi* detected by culture and PCR in clinical relapse of disseminated Lyme borreliosis. Ann. Med..

[54] Berglund J., Stjernberg L., Ornstein K., Tykesson-Joelsson K., Walter H. (2002). Follow-up study of patients with neuroborreliosis. Scand. J. Infect. Dis..

[55] Kaiser R. (2004). Clinical courses of acute and chronic neuroborreliosis following treatment with ceftriaxone. Nervenarzt.

[56] Cameron D. (2008). Severity of Lyme disease with persistent symptoms. Insights from a double-blind placebo-controlled clinical trial. Minerva Med..

[57] Fallon B.A., Keilp J.G., Corbera K.M., Petkova E., Britton C.B., Dwyer E., Slavov I., Cheng J., Dobkin J., Nelson D.R. (2008). A randomized, placebo-controlled trial of repeated IV antibiotic therapy for Lyme encephalopathy. Neurology.

[58] Stricker R.B., Delong A.K., Green C.L., Savely V.R., Chamallas S.N., Johnson L. (2011). Benefit of intravenous antibiotic therapy in patients referred for treatment of neurologic Lyme disease. Int. J. Gen. Med..

[59] Middelveen M.J., McClain S.A., Bandoski C., Israel J.R., Burke J., MacDonald A.B., Timmaraju A., Sapi E., Wang Y., Franco A. (2014). Granulomatous hepatitis associated with chronic *Borrelia burgdorferi* infection: A case report. Res. Open Access..

[60] Shah J.S., Du Cruz I., Narciso W., Lo W., Harris N.S. (2014). Improved sensitivity of Lyme disease Western blots prepared with a mixture of *Borrelia burgdorferi* strains 297 and B31. Chronic Dis. Int..

[61] Middelveen M.J., Bandowski C., Burke J., Sapi E., Filush K.R., Wang Y. (2015). Exploring the association between Morgellons disease and Lyme disease: Identification of *Borrelia burgdorferi* in Morgellons disease patients. BMC Dermatol..

[62] Bankhead T., Chaconas G. (2007). The role of VlsE antigenic variation in the Lyme disease spirochete: Persistence through a mechanism that differs from other pathogens. Mol. Microbiol..

[63] O’Rourke M., Traweger A., Lusa L., Stupica D., Maraspin V., Barrett P.N., Strle F., Livey I. (2013). Quantitative detection of *Borrelia burgdorferi* sensu lato in erythema migrans skin lesions using internally controlled duplex real time PCR. PLoS ONE.

[64] Margos G., Hojgaard A., Lane R.S., Cornet M., Fingerle V., Rudenko N., Ogden N., Aanensen D.M., Fish D., Piesman J. (2010). Multilocus sequence analysis of *Borrelia bissettii* strains from North America reveals a new Borrelia species, *Borrelia kurtenbachii*. Ticks Tick Borne Dis..

[65] Clark K.L., Leydet B., Hartman S. (2013). Lyme borreliosis in human patients in Florida and Georgia, USA. Int. J. Med. Sci..

[66] Sapi E., Pabbati N., Datar A., Davies E.M., Rattelle A., Kuo B.A. (2013). Improved culture conditions for the growth and detection of Borrelia from human serum. Int. J. Med. Sci..

[67] Mayne P.J. (2012). Investigation of *Borrelia burgdorferi* genotypes in Australia obtained from erythema migrans tissue. Clin. Cosmet. Investig. Dermatol..

[68] Mayne P. (2014). Clinical determinants of Lyme borreliosis, babesiosis, bartonellosis, anaplasmosis, and ehrlichiosis in an Australian cohort. Int. J. Gen. Med..

[69] Bunikis J., Tsao J., Garpmo U., Berglund J., Fish D., Barbour A.G. (2004). Sequence typing reveals extensive strain diversity of the Lyme borreliosis agents *Borrelia burgdorferi* in North America and *Borrelia afzelii* in Europe. Microbiology.

[70] Travinsky B., Bunikis J., Barbour A.G. (2010). Geographic differences in genetic locus linkages for *Borrelia burgdorferi*. Emerg. Infect. Dis..

[71] Snydman D.R., Schenkein D.P., Berardi V.P., Lastavica C.C., Pariser K.M. (1986). *Borrelia burgdorferi* in joint fluid in chronic Lyme arthritis. Ann. Intern. Med..

[72] Stanek G., Klein J., Bittner R., Glogar D. (1990). Isolation of *Borrelia burgdorferi* from the myocardium of a patient with longstanding cardiomyopathy. N. Engl. J. Med..

[73] Ma Y., Sturrock A., Weis J.J. (1991). Intracellular localization of *Borrelia burgdorferi* within human endothelial cells. Infect. Immun..

[74] Georgilis K., Peacocke M., Klempner M.S. (1992). Fibroblasts protect the Lyme disease spirochete, *Borrelia burgdorferi*, from ceftriaxone in vitro. J. Infect. Dis..

[75] Haupl T., Hahn G., Rittig M., Krause A., Schoerner C., Schönherr U., Kalden J.R., Burmester G.R. (1993). Persistence of *Borrelia burgdorferi* in ligamentous tissue from a patient with chronic Lyme borreliosis. Arthritis Rheum..

[76] Klempner M.S., Noring R., Rogers R.A. (1993). Invasion of human skin fibroblasts by the Lyme disease spirochete, *Borrelia burgdorferi*. J. Infect. Dis..

[77] Aberer E., Kersten A., Klade H., Poitschek C., Jurecka W. (1996). Heterogeneity of *Borrelia burgdorferi* in the skin. Am. J. Dermatopathol..

[78] Girschick H.J., Huppertz H.I., Rüssmann H., Krenn V., Karch H. (1996). Intracellular persistence of *Borrelia burgdorferi* in human synovial cells. Rheumatol. Int..

[79] Nanagara R., Duray P.H., Schumacher H.R. (1996). Ultrastructural demonstration of spirochetal antigens in synovial uid and synovial membrane in chronic Lyme disease: Possible factors contributing to persistence of organisms. Hum. Pathol..

[80] Hastey C.J., Elsner R.A., Barthold S.W., Baumgarth N. (2012). Delays and diversions mark the development of B cell responses to Borrelia burgdorferi infection. J. Immunol..

[81] Livengood J.A., Gilmore R.D. (2006). Invasion of human neuronal and glial cells by an infectious strain of *Borrelia burgdorferi*. Microbes Infect..

[82] Wormser G.P., Dattwyler R.J., Shapiro E.D., Halperin J.J., Steere A.C., Klempner M.S., Krause P.J., Bakken J.S., Strle F., Stanek G. (2006). The clinical assessment, treatment, and prevention of lyme disease, human granulocytic anaplasmosis, and babesiosis: Clinical practice guidelines by the Infectious Diseases Society of America. Clin. Infect. Dis..

[83] Stricker R.B., Johnson L. (2013). *Borrelia burgdorferi* aggrecanase activity: More evidence for persistent infection in Lyme disease. Front. Cell Infect. Microbiol..

[84] Wu J., Weening E.H., Faske J.B., Höök M., Skare J.T. (2011). Invasion of eukaryotic cells by *Borrelia burgdorferi* requires β(1) integrins and Src kinase activity. Infect. Immun..

[85] Sapi E., Balasubramanian K., Poruri A., Maghsoudlou J.S., Socarras K.M., Timmaraju A.V., Filush K.R., Gupta K., Shaikh S., Theophilus P.A. (2016). Evidence of in vivo existence of Borrelia biofilm in Borrelial lymphocytomas. Eur. J. Microbiol. Immunol..

[86] Feng J., Zhang S., Shi W., Zhang Y. (2016). Ceftriaxone pulse dosing fails to eradicate biofilm-like microcolony *B. burgdorferi* persisters which are sterilized by daptomycin/doxycycline/cefuroxime without pulse dosing. Front. Microbiol..

[87] Klempner M.S., Hu L.T., Evans J., Schmid C.H., Johnson G.M., Trevino R.P., Trevino B.S., DeLona Norton M.P.H., Lois Levy M.S.W., Diane Wall R.N. (2001). Two controlled trials of antibiotic treatment in patients with persistent symptoms and a history of Lyme disease. N. Engl. J. Med..

[88] Wormser G.P., Nadelman R.B., Schwartz I. (2012). The amber theory of Lyme arthritis: Initial description and clinical implications. Clin. Rheumatol..

[89] Aucott J.N. (2015). Post-treatment Lyme disease syndrome. Infect. Dis. Clin. N. Am..

[90] Lazarus J.J., McCarter A.L., Neifer-Sadhwani K., Wooten R.M. (2012). ELISA-based measurement of antibody responses and PCR-based detection profiles can distinguish between active infection and early clearance of *Borrelia burgdorferi*. Clin. Dev. Immunol..

[91] Feng J., Shi W., Zhang S., Zhang Y. (2015). Persister mechanisms in *Borrelia burgdorferi*: Implications for improved intervention. Emerg. Microbes Infect..

[92] Sharma B., Brown A.V., Matluck N.E., Hu L.T., Lewis K. (2015). *Borrelia burgdorferi*, the causative agent of Lyme disease, forms drug-tolerant persister cells. Antimicrob. Agents Chemother..

[93] Cabello F.C., Godfrey H.P., Bugrysheva J., Newman S.A. (2017). Sleeper cells: The stringent response and persistence in the *Borreliella (Borrelia) burgdorferi* enzootic cycle. Environ. Microbiol..

[94] Middelveen M.J., Stricker R.B. (2016). Morgellons disease: A filamentous borrelial dermatitis. Int. J. Gen. Med..

[95] Middelveen M.J., Fesler M.C., Stricker R.B. (2018). History of Morgellons disease: From delusion to definition. Clin. Cosmet. Investig. Dermatol..

[96] Savely V.R., Stricker R.B. (2007). Morgellons disease: The mystery unfolds. Expert Rev. Dermatol..

[97] Savely V.R., Stricker R.B. (2010). Morgellons disease: Analysis of a population with clinically confirmed microscopic subcutaneous fibers of unknown etiology. Clin. Cosmet. Investig. Dermatol..

[98] Middelveen M.J., Stricker R.B. (2011). Filament formation associated with spirochetal infection: A comparative approach to Morgellons disease. Clin. Cosmet. Investig. Dermatol..

[99] Middelveen M.J., Mayne P.J., Kahn D.G., Stricker R.B. (2013). Characterization and evolution of dermal filaments from patients with Morgellons disease. Clin. Cosmet. Investig. Dermatol..

[100] Middelveen M.J., Burugu D., Poruri A., Burke J., Mayne P.J., Sapi E., Kahn D.G., Stricker R.B. (2013). Association of spirochetal infection with Morgellons disease. F1000Res.

[101] Chmielewski T., Tylewska-Wierzbanowska S. (2010). Interactions between *Borrelia burgdorferi* and mouse fibroblasts. Pol. J. Microbiol..

[102] Radolf J.D., Deka R.K., Anand A., Šmajs D., Norgard M.V., Yang X.F. (2016). *Treponema pallidum*, the syphilis spirochete: Making a living as a stealth pathogen. Nat. Rev. Microbiol..

[103] Brorson Ø., Brorson S.H. (1997). Transformation of cystic forms of Borrelia burgdorferi to normal mobile spirochetes. Infection.

[104] Murgia R., Cinco M. (2004). Induction of cystic  forms by different stress conditions in *Borrelia burgdorferi*. APMIS.

[105] MacDonald A.B. (2013). *Borrelia burgdorferi* tissue morphologies and imaging methodologies. Eur. J. Clin. Microbiol. Infect. Dis..

[106] Mestecky J., Moldoveanu Z., Russell M.W. (2005). Immunologic uniqueness of the genital tract: Challenge for vaccine development. Am. J. Reprod. Immunol..

[107] Cu-Uvin S., DeLong A.K., Venkatesh K.K., Hogan J.W., Ingersoll J., Kurpewski J., De Pasquale M.P., D’Aquila R., Caliendo A.M. (2010). Genital tract HIV-1 RNA shedding among women with below detectable plasma viral load. AIDS.

[108] Mzingwane M.L., Tiemessen C.T. (2017). Mechanisms of HIV persistence in HIV reservoirs. Rev. Med. Virol..

[109] Vetter P., Fischer W.A., Schibler M., Jacobs M., Bausch D.G., Kaiser L. (2016). Ebola virus shedding and transmission: Review of current evidence. J. Infect. Dis..

[110] Moreira J., Peixoto T.M., Siqueira A.M., Lamas C.C. (2017). Sexually acquired Zika virus: A systematic review. Clin. Microbiol. Infect..

