# Supplementary material for: Persistent Borrelia Infection in Patients with Ongoing Symptoms of Lyme Disease
Source: Healthcare (Basel). 2018 Apr 14;6(2):33. doi: 10.3390/healthcare6020033 (PMC6023324; doi:10.3390/healthcare6020033)
Supplement: Supplementary file 1 [file healthcare-06-00033-s001.pdf]

Supplemental File: PCR Sequences and BLAST Analyses from Individual Patient Cultures.

UNH, University of New Haven; AB, Australian Biologics; UCI, University of California Irvine.

SEQUENCES FROM UNIVERSITY OF NEW HAVEN

Sequences for Case #1 UNH

Whole callus
pyrG

NNNNNNNNNNNANNNNNNNNNNNNNNNNNGGGAGTGGTAATATTTCTTTTATTCATTTAA
CATATGTGCCAAGTCCAGCTGGAATTAATGAGCAAAAATCTAAACCTACTCAACAAAGTGT
TAAAACCTTAAATAAAGCAGGTATTTTCCCCGATTTAATTATTGCTAGAAGTTCACAAGTAT
TGACAGACCAAATCAGAAAAAAAGTGGCAATGTTTTGCAATGTTGAGAGCACTTCTATTAT
TGACAATGTTGATGTTTCTACTATTTATGAAATTCCTATATCTTTTTATAAGCAGGGTGTACA
TGAGATTTTAAGCTCTAAGTTAAATATTAAGGTTGATCCAAAAATAGAAGAGCTTTCAAAG
CTTGTAGGAGTTATAAAATCTAATTTTTTTGTGCCTAAAAAAATTATTAATATTGCTATTTGT
GGTAAATATGCTGAACTTGATGATTCTTATGCATCAATTAGAGAGTCTTTGGTTCATGTTGC
AGCCCATTTGGATTTGCTTATTAAAAGCACTTTAATTGATTCTAATGATTTAAATGAGAGCT
GTTTAAAAGAGTTTGACGGCATTATTGTTCCTGGCGGCTTTGGAGGCAAAGGATATGAAGG
TAAAATTATGGCTATTAAATATGCTCGTGAGAATAATATTCCCTTTCTTGGAATTTGNCCTTG
NNN

Length 680 bp, BLAST 100% identity with Bbss, e value 0

Whole callus
Fla

NNNNNNNNNNNNNNNNNNNNANNACTNNNNNNNNNCGAAATTAATAGAATTGCTGATC
AAGCTCAATATAACCAAATGCACATGTTATCAAACAAATCTGCTTCTCAAAATGTAAGAAC
AGCTGAAGAGCTTGGAATGCAGCCTGCAAAAATTAACACACCAGCATCACTTTCAGGGTCT
CAAGCGTCTTGGACTTTAAGAGTTCATGTTGGAGCAAACCAAGATGAAGCTATTGCTGTAA
ATATTTATGCAGCTAATGTTGCAAATCTTTTCTCTGGTGAGGGAGCTCAAACTGCTCAGGCT
GCACCGGTTCAAGAGGGTGTTCAACAGGAAGGAGCTCAACAGCCAGCACCTGCTACAGCA
CCTTCA

Length 367 bp, BLAST 100% identity with Bbss, e value 2e-172

Blood culture
Fla

Forward
NNNNNNNNNNNNNANNNNANCAACNNACAGACGAAATTAATAGAATTGCTGATCAAGC
TCAATATAACCAAATGCACATGTTATCAAACAAATCTGCTTCTCAAAATGTAAGAACAGCT
GAAGAGCTTGGAATGCAGCCTGCAAAAATTAACACACCAGCATCACTTTCAGGGTCTCAA
GCGTCTTGGACTTTAAGAGTTCATGTTGGAGCAAACCAAGATGAAGCTATTGCTGTAAATA

TTTATGCAGCTAATGTTGCAAATCTTTTCTCTGGTGAGGGAGCTCAAACTGCTCAGGCTGCA
CCGGTTCAAGAGGGTGTTCAACAGGAAGGAGCTCAACAGCCAGCACCTGCTACAGCACCT
TCA

Length 364 bp, BLAST 99% identity with Bbss, e value 2e-176

Reverse
NNNNNNNNNNNNNNNNNNNANNACTNNNNNNNNNCGAAATTAATAGAATTGCTGATC
AAGCTCAATATAACCAAATGCACATGTTATCAAACAAATCTGCTTCTCAAAATGTAAGAAC
AGCTGAAGAGCTTGGAATGCAGCCTGCAAAAATTAACACACCAGCATCACTTTCAGGGTCT
CAAGCGTCTTGGACTTTAAGAGTTCATGTTGGAGCAAACCAAGATGAAGCTATTGCTGTAA
ATATTTATGCAGCTAATGTTGCAAATCTTTTCTCTGGTGAGGGAGCTCAAACTGCTCAGGCT
GCACCGGTTCAAGAGGGTGTTCAACAGGAAGGAGCTCAACAGCCAGCACCTGCTACAGCA
CCTTCA

Length 367 bp, BLAST100% identity with Bbss, e value 2e-172

Sequences for Case #2 UNH

Vaginal culture
pyrG

Forward
ATAGCCATAATTTTACCTTCNTATCCTTTGCCTCCAAAGCCGCCAGGAACAATAATGCCGTC
AAACTCTTTTAAACAGCTCTCATTTAAATCATTAGAATCAATTAAAGTGCTTTTAATAAGCA
AATCCAAATGGGCTGCAACATGAACCAAAGACTCTCTAATTGATGCATAAGAATCATCAA
GTTCAGCATATTTACCACAAATAGCAATATTAATAATTTTTTTAGGCACAAAAAAATTAGAT
TTTATAACTCCTACAAGCTTTGAAAGCTCTTCTATTTTTGGATCAACCTTAATATTTAACTTA
GAGCTTAAAATCTCATGTACACCCTGCTTATAAAAAGATATAGGAATTTCATAAATAGTAG
AAACATCAACATTGTCAATAATAGAAGTGCTCTCAACATTGCAAAACATTGCCACTTTTTTT
CTGATTTGGTCTGTCAATACTTGTGAACTTCTAGCAATAATTAAATCGGGGAAAATACCTGC
TTTATTTAAGGTTTTAACACTTTGTTGAGTAGGTTTAGATTTTTGCTCATTAATTCCAGCTGGA
CTTGGCACATATGTTAAATGAATAAAAGAAATATTACCACTCCCAATCTCCTGTCTTATTTG
TCTTACTGTCTCAATAAATAAAATATTTTTCCATAT 99% B31 sequence, it has one nucleotide difference

Length 656 bp, BLAST 99% identity with Bbss, e value 0

Reverse
GATTGGGAGTGGTAATATTTCTTTTATTCATTTAACATATGTGCCAAGTCCAGCTGGAATTA
ATGAGCAAAAATCTAAACCTACTCAACAAAGTGTTAAAACCTTAAATAAAGCAGGTATTTT
CCCCGATTTAATTATTGCTAGAAGTTCACAAGTATTGACAGACCAAATCNGAAAAAAAGTG
GCAATGTTTTGCAATGTTGAGAGCACTTCTATTATTGACAATGTTGATGTTTCTACTATTTAT
GAAATTCCTATATCTTTTTATAAGCAGGGTGTACATGAGATTTTAAGCTCTAAGTTAAATAT
TAAGGTTGATCCAAAAATAGAAGAGCTTTCAAAGCTTGTAGGAGTTATAAAATCTAATTTTT
TTGTGCCTAAAAAAATTATTAATATTGCTATTTGTGGTAAATATGCTGAACTTGATGATTCTT
ATGCATCAATTAGAGAGTCTTTGGTTCATGTTGCAGCCCATTTGGATTTGCTTATTAAAAGC
ACTTTAATTGATTCTAATGATTTAAATGAGAGCTGTTTAAAAGAGTTTGACGGCATTATTGTT
CCTGGCGGCTTTGGAGGCAAAGGATATGAAGGTAAAATTATGGCTATTAAATATGCTCGTG
AGAATAATATTCCCTTTCTTGGAATTTGTCTTGGTATAA

Length 659 bp, BLAST 99% identity with Bbss, e value 0

Sequences for Case #3 UNH

Blood culture
16S rRNA

Forward
NNNNNNNNNNNNNNNNNNNNNNNGNNTGTAGCATACATTCAGTGGCGAACGGGTGAG
TAACGCGTGGATGATCTACCTATGAGATGGGGATAACTATTAGAAATAGTAGCTAATACCG
AATAAGGTCAGTTAATTTGTTAATTGATGAAAGGAAGCCTTTAAAGCTTCGCTTGTAGATGA
GTCTGCGTCTTATTAGCTAGTTGGTAGGGTAAATGCCTACCAAGGCAATGATAAGTAACCG
GCCTGAGAGGGTGAACGGTCACACTGGAACTGAGATACGGTCCAGACTCCTACGGGAGGC
AGCAGCTAAGAATCTTCCGCAATGGGCGAAAGCCTGACGGAGCGACACTGCGTGAATGAA
GAAGGTCGAAAGATTGTAAAATTCTTTTATAAATGAGGAATAAGCTTTGTAGGA

Length 415 bp, BLAST 99% identity with Bbss, e value 0

Reverse
NNNNNNNNNNNNNNNNNNNNNNNTCTTCNNNCCNCNGNNGNGCGCTCCGTCAGGCTTTC
GCCCATTGCGGAAGATTCTTAGCTGCTGCCTCCCGTAGGAGTCTGGACCGTATCTCAGTTCC
AGTGTGACCGTTCACCCTCTCAGGCCGGTTACTTATCATTGCCTTGGTAGGCATTTACCCTAC
CAACTAGCTAATAAGACGCAGACTCATCTACAAGCGAAGCTTTAAAGGCTTCCTTTCATCA
ATTAACAAATTAACTGACCTTATTCGGTATTAGCTACTATTTCTAATAGTTATCCCCATCTCA
TAGGTAGATCATCCACGCGTTACTCACCCGTTCGCCACTGAATGTATTGCTACATCCCGTTT
GACTTGCATGCTTAAGACGCACTGCCAGCGTTAGTTCTAAGCCAGGA

Length 415 bp, BLAST 100% identity with Bbss, e value 0

Seminal culture
16S rRNA

GTAGCATACATTCAGTGGCGAACGGGTGAGTAACGCGTGGATGATCTACCTATGAGATGGG
GATAACTATTAGAAATAGTAGCTAATACCGAATAAGGTCAGTTAATTTGTTAATTGATGAA
AGGAAGCCTTTAAAGCTTCGCTTGTAGATGAGTCTGCGTCTTATTAGCTAGTTGGTAGGGTA
AATGCCTACCAAGGCAATGATAAGTAACCGGCCTGAGAGGGTGAACGGTCACACTGGAAC
TGAGATACGGTCCAGACTCCTACGGGAGGCAGCAGCTAAGAATCTTCCGCAATGGGCGAA
AGCCTGACGGAGCGACACTGCGTGAATGAAGAAGGTCGAAAGATTGTAAAATTCTTTTATA
AATGAGGAATAAGCTTTGTAGGA

Length 388 bp, BLAST 99% identity with Bbss, e value 0

Sequences for Case #7 UNH

Vaginal culture
16S rRNA

NNNNNGNNNNNNNGCNNNCTTCNGTGGCGACGGGTGAGTAACGCGTGGATGATCTACCTA
TGAGATGGGGATAACTATTAGAAATAGTAGCTAATACCGAATAAGGTCAGTTAATTTGTTA
ATTGATGAAAGGAAGCCTTTAAAGCTTCGCTTGTAGATGAGTCTGCGTCTTATTAGCTAGTT
GGTAGGGTAAATGCCTACCAAGGCAATGATAAGTAACCGGCCTGAGAGGGTGAACGGTCA
CACTGGAACTGAGATACGGTCCAGACTCCTACGGGAGGCAGCAGCTAAGAATCTTCCGCA
ATGGGCGAAAGCCTGACGGAGCGACACTGCGTGAATGAAGAAGGTCGAAAGATTGTAAA
ATTCTTTTATAAATGAGGAATAGCCTTTTGTAGGA

Length 396 bp, BLAST 99% identity with Bbss, e value 0

Sequences for Case #8 UNH

Seminal culture
16S rRNA

AGCTTTGTAGGACCTGGCTTAGAACTAACGCTGGCAGTGCGTCTTAAGCATGAGGAATAAG
CTTTGTAGGACCTGGCTTAGAACTAACGCTGGCAGTGCGTCTTAAGCATGAGGAATAAGCT
TTGTAGGACCTGGCTTAGAACTAACGCTGGCAGTGCGTCTTAAGCATGAGGAATAAGCTTT
GTAGGACCTGGCTTAGAACTAACGCTGGCAGTGCGTCT

Length 221 bp, BLAST 100% identity with Bbss, e value 7e-10

Sequences for Case #12 UNH

Whole callus
uvrA

Forward
CNNNNNNNNNNNNNNNANNGNNTATCAGGTAGTCTATCTGGTGGCGAGGCTCAGCGTA
TTAGGCTTGCTACTCAAATAGGATCAGCACTTTCGGGTGTTATTTATGTTCTTGATGAGCCA
AGTATTGGTCTTCATCAAAGAGATAATGAAAAATTAATCTCTACTCTTGTTAATCTTAAAAA
TCTTGGTAATACTGTAATTGTTGTTGAACATGATGAGCAAACTTTGCGTACTGCGGACTATA
TTATTGATATGGGTCCTGGTGCTGGAATTCTTGGAGGGGAAATAGTTGCAAAGGGAGCCTT
GATTGATATTTTAAATAGCAAAAATAGTTTAACTGGTCAATATCTTAGCGGCAAGTTTAAAA
TAGATGTTCCAAGCTCTAGAAGAAAGGCAGATAAGGGAGAAATTTTGCTTTTGGGCTCTAA
TAAAAATAATCTTAAAAATATAGACTTAAGTATCCCTTTGGGAGTTTTTACCGTAATAACAG
GTGTTTCTGGTAGCGGAAAAAGTACTTTACTTAACGAGGTGTTATATCCAGCTCTTGATAGT
AGATTAAAGCTTAATGAAAAGTATTGTGATGGCTTTAAAGATATTGTTGGGTATGAAAAAA
TCGATAAAATTATTCAAATAAATCAAAAACCCCAATAGGA

Length 653 bp, BLAST 99% identity with Bbss, e value 0

Reverse
NNNNNNNANTNNNNCNACNNNNNNNATATCTTTAAAGCCATCACAATACTTTTCATTAAG
CTTTAATCTACTATCAAGAGCTGGATATAACACCTCGTTAAGTAAAGTACTTTTTCCGCTAC
CAGAAACACCTGTTATTACGGTAAAAACTCCCAAAGGGATACTTAAGTCTATATTTTTAAG
ATTATTTTTATTAGAGCCCAAAAGCAAAATTTCTCCCTTATCTGCCTTTCTTCTAGAGCTTGG
AACATCTATTTTAAACTTGCCGCTAAGATATTGACCAGTTAAACTATTTTTGCTATTTAAAAT
ATCAATCAAGGCTCCCTTTGCAACTATTTCCCCTCCAAGAATTCCAGCACCAGGACCCATAT
CAATAATATAGTCCGCAGTACGCAAAGTTTGCTCATCATGTTCAACAACAATTACAGTATT
ACCAAGATTTTTAAGATTAACAAGAGTAGAGATTAATTTTTCATTATCTCTTTGATGAAGAC
CAATACTTGGCTCATCAAGAACATAAATAACACCCGAAAGTGCTGATCCTATTTGAGTAGC
AAGCCTAATACGCTGAGCCTCGCCACCAGATAGACTACCTGATATTCTATTTAAATATAAA
TAAGAAAGGCCAACATCAATTAANANTTTTNNNGCN

Length 651 bp, BLAST 99% identity with Bbss, e value 0

SEQUENCES FROM AUSTRALIAN BIOLOGICS

Sequences for Case #2 AB

Vaginal culture
rpoC

GGAGATGTTGTAAAAGCAGGAGATATGCTTTGTGATGGTAGAATTAATCCTCATGATGTGC
TTGAAATTTTAGGTGGGA

Length 79 bp, BLAST 100% identity with Bbss, e value 4e-032

Sequences for Case #3 AB

Blood culture
rpoC

TAAAGGGTACTCTCTAGCCTTTTACCTTTTTGATTAGACTCCCACCTAAAATTTCAAGCAGG
GGATTCTACATCTACTAAGCCTTTTACCTTTTTGAATTGAA

Length 103bp, BLAST 96% identity with Bbss, e value 0.11

Seminal culture, 1 month on abx
rpoC
ATTTCTGCAGTGACATCCATGCTATCTCCTGCTTTTACAACATCTCCATCTCTAACCAAAAG
ATGTTTTCCAGCTGGAATATAATGCTTATGTTCAACCCCATACTCATCTAAAATATTAATAA
GCCTTTTACCTTTTTGAATTGA
Length 146bp, BLAST 100% identity with Bbss, e value 9e-57
Seminal culture, 4 months on abx
rpoC
TTAAGAGGCAGCAGCACGTGTATGAGTGTGAATACATAAGTGTTATATTCCAGCTGGAAAA
CATCTTTTGGTTAGAGACGGAGATGTTGTAAAAGCAGGAGATATGCTTTGTGATGGTAGAA
TTAATCCTCATGATGTGCTTGAAATTTTAGGTGGGA
Length 158bp, BLAST 98% identity with Bbss, e value 3e-52

Sequences for Case #4 AB

Vaginal culture
rpoC

TGCTTTTACAACATCTCCGTCTCTAACCAAAAGATGTTTTCCAGCTGGAATATAATGCTTAT
GTTCAACCCCATACTCATCTAAAATATTAATAAGCCTTTTACCTTTTTGAATTGAA

Length 118bp, BLAST 99% identity with Bbss, e value 1e-51

Sequences for Case #5 AB

Vaginal culture
rpoC

TCCAGCTGGAAAACATCTTTTGGTTAGAGATGGAGATGTTGTAAAAGCAGGAGATATGCTT
TGTGATGGTAGAATTAATCCTATGATGTGCTTGAAATTTTAGGTGGGA

Length 109 bp, BLAST 99% identity with Bbss, e value 6e-47

Sequences for Case #11 AB

Vaginal culture
rpoC

TATGGTAATGATCATGTGTATGAGGTAGGACGGTGGCGATGATTGTCCTTTTAGGTGGCAAG
GTGGGATGCTTGATGTTTTTGGTGGAAAAGCAGGAGATATGCTTTGTGATGGTAGAATTAAT
CCTCATGATGTGCTTGAAATTTTAGGTGGGAA

Length 156 Bp, BLAST 100% identity with Bbss, e value 1e-25

SEQUENCES FROM UNIVERSITY OF CALIFORNIA, IRVINE

Sequences for Case #2 UCI

16S-23S Intergenic spacer
Vaginal culture

ATGCGTTTTGCTTCACATCACTGTTTCGCTTCGCTTTGTACAGGCCATTGTAGCACGTGTGTA
GCCCAGGACATAAGGGCCATGATGATTTGACGTCATCCTCACCTTCCTCCGACTTATCACCG
GCAGTCTTATCTGAGTCCCCACCATTACATGCTGGTAACAGATAACAAGGGTTGCGCTCGTT
GCGGGACTTAACCCAACACCTCACAGCACGAGCTGACGACAACCATGCAGCACCTGTATA
TAGACCCCAAACGGGGAATAATTATCTCTAACTATATCCTATATATGTCAAGCCCTGGTAA
GGTTCCTCGCGTATCATCGAATTAAACCACATGCTCCACCGCTTGTGCGGGCCCCCGTCAAT
TCCTTTGAGTTTCACTCTTGCGAGCATACTCCCCAGGCGGCACACTTAACACGTTAGCTTCG
GTACTAACTTTTAGTTAACACCAAGTGTGCATCGTTTACAGC

Length 474bp, BLAST 100% identity with Bbss, e value 0

Sequences for Case #10 UCI

16S-23S Intergenic spacer
Seminal culture

ATGCGTTTTGCTTCACATCACTGTTTCGCTTCGCTTTGTACAGGCCATTGTAGCACGTGTGTA
GCCCAGGACATAAGGGCCATGATGATTTGACGTCATCCTCACCTTCCTCCGACTTATCACCG
GCAGTCTTATCTGAGTCCCCACCATTACATGCTGGTAACAGATAACAAGGGTTGCGCTCGTT
GCGGGACTTAACCCAACACCTCACAGCACGAGCTGACGACAACCATGCAGCACCTGTATA
TAGACCCCAAACGGGGGATAATTATCTCTAACTATATCCTATATATGTCAAGCCCTGGTAA
GGTTCCTCGCGTATCATCGAATTAAACCACATGCTCCACCGCTTGTGCGGGCCCCCGTCAAT
TCCTTTGAGTTTCACTCTTGCGGGCATACTCCCCAGGCGGCACACTTAACACGTTAGCCTCG
GTACTAACTTTTAGTTAACACCAAGTGTGCATCGTTTACAGC

Length 474bp, BLAST 99% identity with Bbss, e value 0
